# Context-Based Filtering for Assisted Brain-Actuated Wheelchair Driving

**DOI:** 10.1155/2007/25130

**Published:** 2007-07-26

**Authors:** Gerolf Vanacker, José del R. Millán, Eileen Lew, Pierre W. Ferrez, Ferran Galán Moles, Johan Philips, Hendrik Van Brussel, Marnix Nuttin

**Affiliations:** ^1^The Department of Mechanical Engineering, Katholieke Universiteit, 3001 Leuven , Belgium; ^2^The IDIAP Research Institute, 1920 Martigny , Switzerland

## Abstract

Controlling a robotic device by using human brain signals is an interesting and challenging task. The device may be complicated to control and the nonstationary nature of the brain signals provides for a rather unstable input. With the use of intelligent processing algorithms adapted to the task at hand, however, the performance can be increased. This paper introduces a shared control system that helps the subject in driving an intelligent wheelchair with a noninvasive brain interface. The subject's steering intentions are estimated from electroencephalogram (EEG) signals and passed through to the shared control system before being sent to the wheelchair motors. Experimental results show a possibility for significant improvement in the overall driving performance when using the shared control system compared to driving without it. These results have been obtained with 2 healthy subjects during their first day of training with the brain-actuated wheelchair.

## 1. INTRODUCTION

The continuing progress in the research for noninvasive BCI classification systems gives rise to a wealth of potential practical applications. The prospect of humans interfacing the mechanical world through brain-coupled devices and thereby controlling everyday machines through the process of mere thought is certainly an appealing one as discussed in [1–3]. A promising class of applications are those concerning assistive devices for people with serious impairments. The classical interfaces that disabled people commonly used to control or manipulate an assistive device typically require the patient to have adequate control over one or more physical components of his or her body. Typically, that would be one of the limbs: an arm, hand, or finger. Bioprosthetic systems that are controlled directly through brain signals on the other hand could provide for a more natural extention of human capabilities. Especially in the case where the patient is completely paralysed, this technology may provide for the only possible way for him/her to gain control over basic aspects of his/her daily life.

**Figure 1:**
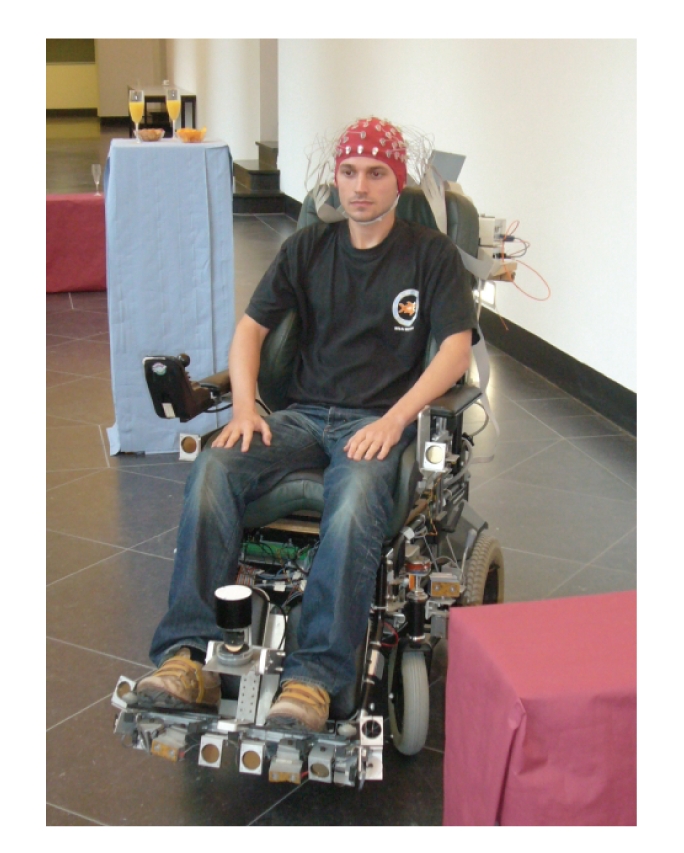
A subject controlling our robotic platform Sharioto in a natural indoor environment through noninvase EEG. Visible are the sensors of the platform: a laser range scanner in front and sonar sensors all around.

Amongst these, the ability to control the personal mobility is generally considered an important one. The reduction in mobility that many people experience, due to various impairments or simply due to the effects of ageing, often has a profound impact on the person's independence, social activity, and self-esteem. For many people suffering from a diverse range of impairments, the primary device that could provide for that mobility is the electrical wheelchair. It is worth noting, however, that in case of locked-in patients their highest priority is not mobility. Still, learning how to make it possible to drive complex devices such a wheelchair will also lead to better communication and domotic tools. Many patients, however, do not have the ability to exercise the demanding fine control that wheelchair steering requires, even with an input device capable of communicating a high level of detail, such as the classical joystick. Problems regarding not only the physical inability to accurately manipulate the joystick, but also a reduced kinematical and dynamical insight in the wheelchair motion regularly occur, as was seen in earlier work [4]. Therefore, the prospect of wheelchair control through a brain-coupled control interface, which is in general less reliable than a classical interface, may seem a remote one. Nevertheless, recent results have shown the feasibility of such brain-actuated wheelchairs; see Figure 1 and [1].

Over the past years, important advances in research concerning *shared control* techniques have been made, as may be seen in [4–7]. Shared control systems typically feature one or more intelligent algorithms that aim at assisting the human to execute some task at hand. Both human(s) and intelligent controller(s) then *share* the control over a device whereby each of the actors may exercise influence through the manipulation of some control variables. Together, through cooperative behavior, they aim at completing the task in a way which is hoped to be superior to the situation where only a single actor is in control. In the specific case of assisted wheelchair driving, the actors are the patient and an intelligent controller. The variables to be shared are the translational and rotational velocity of the robot (*v*,ω). Also, in this class of applications, the human typically has *supervisory* control, meaning that it is him or her that defines the *global plan* that has to be executed. The other actors then need to adopt this plan and cooperate accordingly. Furthermore, an intelligent actor cooperating in a shared control system that is designed to operate with a brain computer interface (BCI) as the human input needs to accommodate for the specific properties that this particular input has. 

This paper presents a shared control system for use with a brain computer interface (BCI). The intelligent controller is designed to *filter* out the possible erroneous mental commands inferred by the BCI from noninvasive electroencephalogram (EEG) signals. It estimates the environmental *context* and uses that to detect illogical steering signals, according to the intention —the global plan —the human has. In the proposed framework, the patient has *continuous* control over the wheelchair, parallel to classical joystick control. This allows for a more natural interaction with the robotic assistant, as well as *fine motion* control. The organization of this paper is as follows. In Section 2, we will briefly discuss related work in shared control techniques for wheelchair navigation. Section 3 introduces our new shared control system based on context estimation and signal filtering. In Section 4, we then present experimental results that validate this approach. Finally, Sections 5 and 6 present, respectively, a discussion of the results and the general conclusions of this work.

The brain-actuated wheelchair described in this paper is an extension of the brain-actuated mobile robot developed by Millán et al. [1]. In this paper, we focus on the main innovation of such first prototype, namely, novel features of the shared control framework specifically designed to work with a BCI. Details of the BCI can be found in [1].

## 2. RELATED WORK

In the past years, a fair number of research groups have ventured into the search for shared control techniques in order to provide assistance to patients as they experience problems when driving an electrical wheelchair. Because of the many different types of manoeuvres that may induce driving problems, for example, driving through a door, obstacle avoidance, driving in a small corridor, docking at a table and others, different algorithms have been developed to cope with these specific situations. This led to the fact that most of the existing approaches focus on the development and selection of such discrete *modes* . Roughly speaking, one may divide the approaches in those that require the user to *explicitly* choose the mode [8–12] on the one hand and those that provide automatic—*implicit*—mode changes based on an interpretation of the surroundings and the user input [6, 7, 13, 14].

Not only the latter group of approaches provide for a more natural interaction between patient and robot, but automatic mode changes are also necessary for a group of patients that are physically unable to communicate their choice on the provided interface. Consider, for example, an array of buttons, each of which activates another assistance mode. A patient suffering from, for instance, multiple sclerosis might experience large difficulties to accurately reach and press the wanted button. The central problem in these implicit approaches therefore is the question: “What is the user's intention?” [6, 7]. Research addressing that question is performed at the *Mobile Learning Robot* (MLR) research group of the Department of Mechanical Engineering at the K. U. Leuven.^1^ Another approach centres on establishing a relation between the steering commands that a capable able-bodied user would give—the so-called *reference* signals—and the signals of the specific patient, given the same situation and the same global intention, introduced in [5]. Knowledge over both allows for a conversion of the less than optimal patient steering signals to the optimal reference signals, thereby *filtering* out the steering handicap.

A similar technique filtering may be used to improve the driving performance of a BCI-controlled wheelchair, keeping in mind the specifics of this particular interface. In comparison with the classical analog joystick, as used in [5], the BCI input generally has a limited resolution and higher uncertainty.

## 3. APPROACH

**Figure 2:**
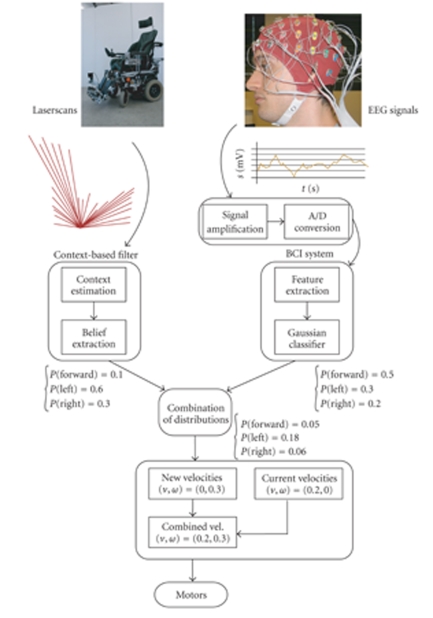
A schematic diagram showing the flow of information in the system. On the left-hand side, environmental information from the wheelchair's sensors (the laser range scanner) feeds the contextual filter that builds a probability distribution over the possible (local) user steering actions. On the right-hand side, the EEG data is fed into the BCI system that estimates the probability of the different mental commands. Both streams of information are combined to form a filtered estimate of the user's steering intent which is eventually sent to the wheelchair's motors as explained in Section 3.1.

This paper presents an assistive algorithm specifically designed to help a BCI subject navigate an electrical wheelchair in an everyday environment. It uses an estimate of the environmental *context* to build a probability distribution over the possible steering commands and uses that information to “filter” out possible erroneous user signals. The hypothesis is that with this assistance, the overall driving performance will improve, especially for “novel” subjects, that is, subjects with little or no former experience in BCI control. Figure 2 illustrates the general architecture of the brain-actuated wheelchair.

### 3.1. BCI-generated commands and interpretation

The nature of BCI-classified mental commands, generated by the subject to indicate some desired movement is quite different from those generated by a continuous joystick. First and foremost, there is an important reduction in resolution due to the limited amount of different mental commands that a BCI classifier can reliably discern. As a consequence, a command-to-movement scheme must be adopted which ensures that smooth motion will result from these discrete input signals. The EEG classifier system used in this work (see [1]) is able to distinguish three discrete commands that may express the need for movement into a certain direction. The steering signals that the classifier outputs consist of a probability distribution over these three discrete steering commands: *Forward* , *Left* , and *Right* . In order to provide intuitive control, we would like to enable the patient to exercise *velocity control* over the platform, so the probability distribution expresses the BCI's belief about the intent of the user to alter the current *velocity* of the wheelchair. *Forward* means that the translational speed *v* should be increased or maintained— when the maximum speed is already reached. A *Left* or *Right* signal means that the user intends to rotate the wheelchair in the respective direction, thus increasing or decreasing the rotational velocity *ω*. Both velocities are superimposed, so that a command to turn when the wheelchair is already moving forward will result in a smoothly curved path.

To accommodate for smooth motion, the system maintains the translational speed for a number of seconds, so that the human does not have to constantly generate *Forward* commands when driving straight on. This also prevents the robot from coming to a halt when taking turns. When for a certain period no *Forward* command is issued, however, the robot does effectively stop. For similar reasons, a signal that triggers a rotational command is only executed for a small amount of time. This prevents that the platform keeps turning for too long and overshoots the direction in which the subject intended to continue his travel.

### 3.2. Context

In typical everyday life, a wheelchair user may come to face a large number of different situations. The nature of a situation is primarily dependent on the environmental settings. Together with the *intention* (the plan) of the user, this environmental situation is part of the *context* in which the controller needs to operate. The assistive system should be able to provide help in as many of these contexts as possible. Because of the different nature of each situation, the controller should be able to detect the specific type of context at hand automatically if it is to help the human in an appropriate manner.

#### 3.2.1. Estimating the context

**Figure 3:**
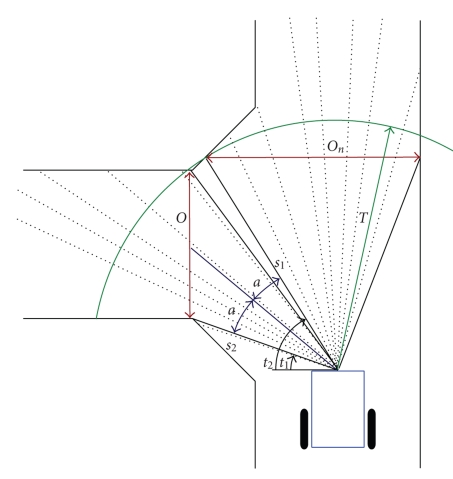
The principle of the context estimator. With a laser range scanner, a set of regions is detected that provide safe manoeuvrable openings in the environment. The number and location of these openings, together with the intention of the human, then provides the context. The figure shows how the region to the left and the one in front of the robot are detected as openings.

For this work, context estimation was done by defining a general, a priori-known user intention (smooth and efficient forward navigation through the environment) on the one hand and a constant automatic estimation of the environmental situation on the other hand. The situations were modelled as the number and location of *openings*: wide, open spaces to which the user might safely navigate. The principle is as follows: suppose the wheelchair is approaching a crossroad, as depicted in Figure 3. The laser scanner in front of the wheelchair scans 180 degrees and senses the distance to the environment for every degree. The algorithm then searches for regions with consecutive scans for which the distance is larger than a certain threshold *T*. This results in a number of regions that qualify as candidates for an opening. Next, for each of the resulting regions, the width of the opening *O* is calculated:

(1) $$O=\sqrt{s_{1}^{2}+s_{2}^{2}-2s_{1}s_{2}\cos(t_{2}-t_{1})}.$$

This length is then compared to the physical dimensions of the wheelchair (its width). If the length *O* exceeds the wheelchair width augmented with a safety margin, the corresponding region is accepted as an opening. Its orientation with respect to the current wheelchair position is then π/2 − (*t*_2_ − *t*_1_)/2.

#### 3.2.2. Extracting a belief in user actions

Each opening then represents a general direction in which the user might opt to continue his travel. With this knowledge about the current situation, a probability distribution concerning the possible *local* user actions may be built. Note that inferring these probabilities requires the knowledge of the global intention of the human. In this case, it is supposed that the user wishes to navigate safely and efficiently through the environment without halting or going backwards. In other cases, a user might also wish to stop at certain locations, or dock at particular places.

**Figure 4:**
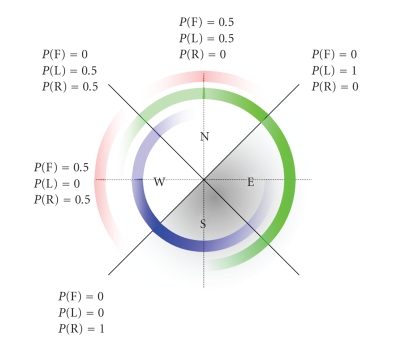
Extracting beliefs from the context in function of the wheelchair orientation. Four quadrants are shown, representing a situation in which possible directions are arranged orthogonal. The inner circle shows the probability of a *Right* command, the middle circle the probability of a *Left* command, and the outer circle the probability of a *Forward* command.

When the directions in which the robot can travel are orthogonal, as in Figure 3, we can summarize the environmental belief in four quadrants, as depicted in Figure 4. The figure shows how the regions West and North are deemed probable navigation directions, as extracted from the environment (see Figure 3). The regions East and South, on the other hand, are improbable (as the scanner sees a wall on the right hand, and going backwards is also not probable given the intention of smooth forward navigation). If the wheelchair is oriented North, the controller attaches a probability of 0.5 to *Forward* and *Left* . *P*_env_ (*Right*) is set to zero, because rotating to the right would make the robot turn towards an obstacle (the wall). The possibility of turning into the corridor to the left is reflected in *P*_env_ (*Left*) = 0.5. If the wheelchair is oriented 45 degrees North-West, *P*_env_ (*Forward*) has become zero, while the possible commands now are *Left* and *Right* , with equal probability, reflecting the belief that one of the orthogonal directions North or West should be chosen. When the wheelchair is turning further towards West, *Forward* becomes possible again, and *P*_env_ (*Right*) stays constant while *P*_env_ (*Left*) diminishes completely. At the boundary between the probable directions and those that are improbable, the controller attaches a maximum belief to those commands that would keep the wheelchair in the half plane of high probability. Between the above-described orientations, the probabilities are interpolated linearly. This is depicted in Figure 4 as the linearly changing transparency of the respective circle.

#### 3.2.3. Combining the beliefs

The intelligent controller now needs to combine the signals coming from the EEG classifier with the probability distribution generated from the environmental knowledge, so as to get a better estimation of the user's local steering intent. Different ways of combining the probabilities from EEG classifier and environment may be chosen [15]. In this work, the *product* operator was used, mainly because the classifier can occasionally attribute a high probability to the wrong class, and averaging the contributions of EEG classifier and environment may still lead to a fairly high probability for a command that is in fact very unlikely. Using the product in this case yields more likely combinations. The resulting probability for a certain class *C* thus becomes

(2) *P*(*C*) = *P*_EEG_(*C*) ⋅ 
*P*_env_(*C*).

From the resulting distribution, the command with the *highest* probability is selected and applied to the wheelchair motors.

## 4. EXPERIMENTS AND RESULTS

### 4.1. Setup

**Figure 5:**
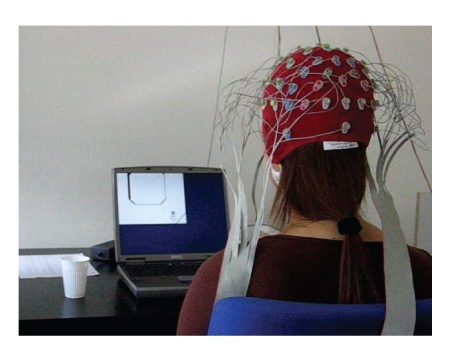
A subject controlling an intelligent wheelchair in a simulated environment. Visible is the EEG sensor cap with the cables that are connected to the BCI system and the computer that runs the shared control system.

**Figure 6:**
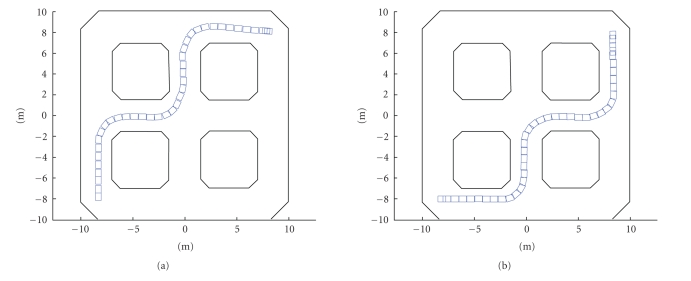
The environment in which the experiments were performed. The wheelchair's position is depicted as a small rectangle at consecutive time steps. Both the paths that the subjects were instructed to follow are shown. It is also worth noting that the initial orientation for each of the paths is different.

Experiments were conducted with a commercially available EEG system feeding the data to the BCI that estimates the user's mental commands. The classifier uses power spectrum information computed from the EEG as its input and outputs the estimated probability distribution over the classes *Left* , *Forward* , and *Right* at a rate of 2 Hz. A second computer running the shared control system is attached to the classifier system and uses its output to control the wheelchair. In this work, a simulated environment was used (mainly for safety reasons) in which a wheelchair was modelled featuring a laser range scanner in front capable of scanning 180 degrees (1 scan for each degree) at 5 Hz. The maximum range of this scanner was fixed to 4.5 m, in accordance with the real physical scanner on our platform Sharioto. The wheelchair was placed in the environment shown in Figure 6. The figure also shows the two paths the subjects were asked to follow. Figure 5 shows a subject during one of the sessions.

Furthermore, because of the inherent nonstationary nature of EEG data, a mild form of online learning was used in the EEG classifier system to continually track the subject's brain signals [16].

### 4.2. Experimental design

For the experiments, two able-bodied voluntary subjects were asked to control the wheelchair for a large number of sessions spanning over several days. This not only allowed to test the performance of the proposed shared control system, but also the *evolution* of the subject's control with and without filter. In between the sessions, the filter was occasionally (de-)activated without the subject's knowledge to investigate the effects of mental model switches and phenomena such as mode confusion [14]. Both subjects were novel with respect to BCI control as well as control of an electrical wheelchair. On the first day we asked the subjects to simply control the wheelchair regardless of any goals in the map, allowing them to get accustomed to using the system. On days 2 through 5, the subjects were instructed to follow a path to reach a certain goal position (see Figure 6). While driving, the subject continuously expressed his/her intended direction orally, allowing logging and comparison. When the wheelchair came too close to an obstacle (a wall), obstacle avoidance (OA, see [4] for details) was activated, to prevent the robot from getting stuck. Finally, the subject was allowed to take resting points while driving (simply because BCI control requires deep concentration which cannot be endured for long periods). When the user calls out “stop,” the classifier is paused and no new steering commands are generated. The robot will continue the path it is currently following while the shared control system (obstacle avoidance in this case) would lead it safely away from obstacles, if necessary. For the interpretation of the BCI commands, the following scheme was used:

(3) $$\begin{array}{ll} v_{\text{inc}} & =0.5\;\text{m}/\text{s},\\ \omega_{\text{inc}} & =0.2\;\text{rad}/\text{s},\\ v_{\max} & =1\;\text{m}/\text{s},\\ \omega_{\max} & =0.6\;\text{rad}/\text{s},\\ v_{\text{new}} & =(\begin{array}{cc} \max\{v_{\text{curr}}+v_{\text{inc}},v_{\max}\} & \text{if}\delta t_{v}<10s,\\ 0 & \text{if otherwise}, \end{array}\\ \omega_{\text{new}} & =(\begin{array}{cc} \max\{\omega_{\text{curr}}\pm \omega_{\text{inc}},\omega_{\max}\} & \text{if}\delta t_{\omega }<1s,\\ 0 & \text{if otherwise}, \end{array} \end{array}$$

where δ*t_v_* and δ*t_ω_* are the number of seconds since the last received command for, respectively, translational and rotational motion.

### 4.3. Results

Data was gathered on two distinct levels. First, every command sent by the classifier was logged, as well as the intent of the subject at that time. This allows to compare the output of the classifier with the actual intention of the human on the *individual command* level. Second, when driving towards the goal position, global measures such as the total time needed, the total distance travelled, and the percentage of the time that obstacle avoidance was active were logged to quantify *task performance* .

#### 4.3.1. Individual command level

When comparing the number of times that the intended direction (*Forward* , *Left* , *Right*) was deemed the most likely one by the EEG classifier (attaching it the highest probability), subject 1 showed an overall increase in performance over the course of the five days (from 57.24% on day 1 to 63.98% on day 5). It has to be noted in this respect that this subject was completely new to BCI control as well as wheelchair control. The witnessed evolution may thus be attributed to the human gradually learning to control the system. Subject 2 shows a similar improvement over the first days, from 46.61% on day 1 to 63.14% on day 3 (although performance declines afterwards).

**Figure 7:**
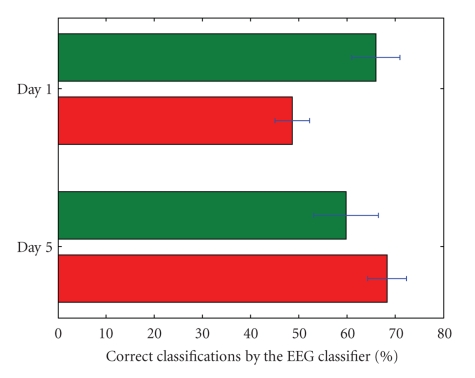
The EEG classifier performance for days 1 and 5 for subject 1. The lower bar in each day depicts the performance when driving without filter, the upper one shows the performance for sessions when filtering was active.

**Figure 8:**
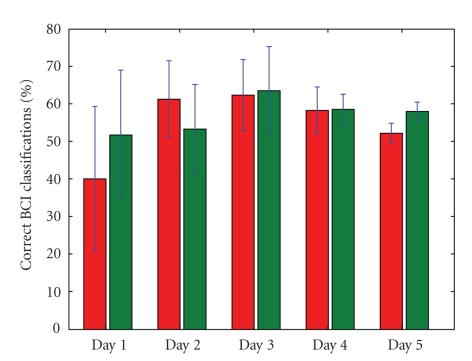
The EEG classifier performance for all days for subject 2. The left bar in each day depicts the performance when driving without filter, the right one shows the performance for sessions when filtering was active.

**Figure 9:**
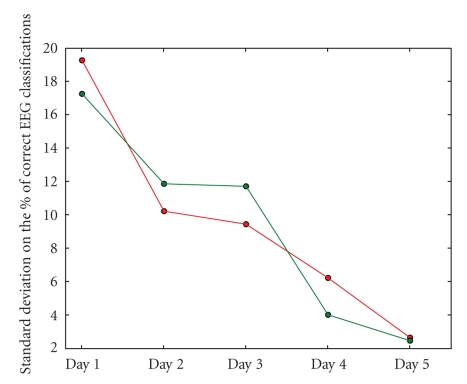
The standard deviations on the EEG classifier performance during the five days for subject 2; sessions with and without filter are shown in green and red, respectively. We can see that the subject shows a more constant performance over the sessions during one day as his experience with controlling the system develops.

For both subjects the classifier performance is different when controlling with or without the environmental filter as is visible in Figures 7 and 8. When the overall BCI performance is rather bad, it is much better to drive with the filter (e.g., subject 1, day 1). On the other hand, when the BCI performance is exceptionally good, driving with the shared control system may make it worse (e.g., subject 1, day 5). It is also worth mentioning that although subject 2 did not show the same increase in average classifier performance over all days (see Figure 8), he showed a steady improvement regarding the standard deviation on the performance (depicted in Figure 9). This reflects the gradually more constant driving behavior of the subject, as his mental driving models become more mature.

**Figure 10:**
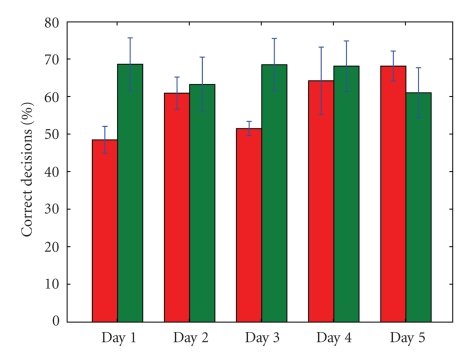
The percentage of control variables (*v*, *ω*) that is in accordance with the human's intention, for subject 1. On the left for each day, we see the performance without environmental filtering. On the right, the results when filtering is active. Also shown are the standard deviations.

**Figure 11:**
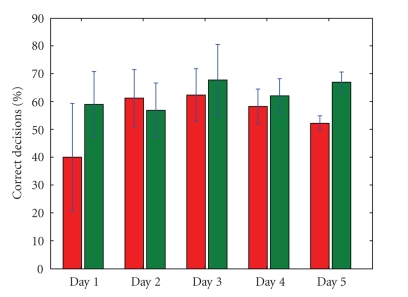
The percentage of control variables (*v*, *ω*) that is in accordance with the human's intention, for subject 2. On the left for each day, we see the performance without environmental filtering. On the right, the results when filtering is active. Also shown are the standard deviations.

**Figure 12:**
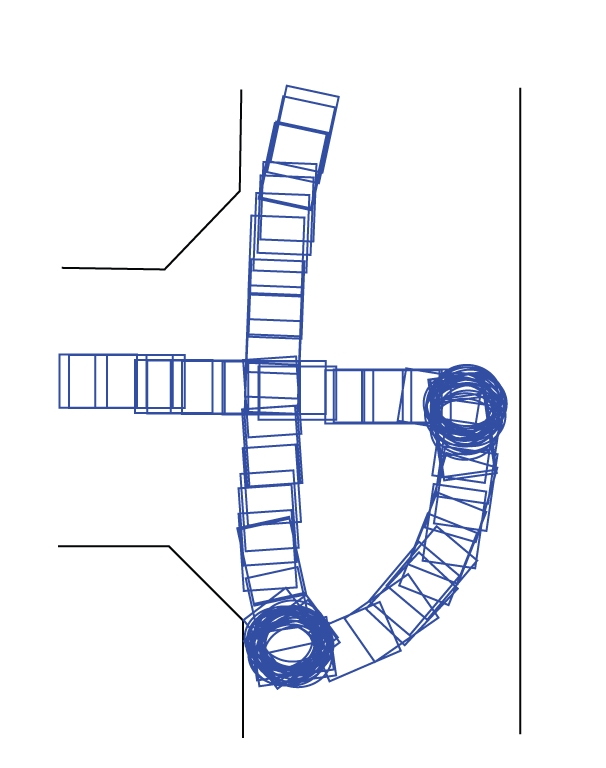
A detail of one trajectory followed by subject 2 on day 2. We can see that the subject tries to turn 180 degrees in a corridor, behavior which is deemed unlikely by the environmental filter.

A similar picture is visible when we look at the actual resulting number of correct *decisions* that were sent to the wheelchair motors (the number of times that the speeds sent to the motors were in accordance with the subject's intent). Without filtering, this number equals that of the “raw” classifier performance. When environmental filtering is used, we get significantly more correct classifications if the EEG signal in itself is rather bad, but we can see that if the BCI performance gets very good (subject 1, day 5 and subject 2, day 2), the filter may actually deteriorate the percentage of correctly executed decisions (see Figures 10 and 11). We may conclude that if there is ample room for improvement (because of a bad EEG signal), the filter improves the situation. Whenever the human (and classifier) perform very well, however, the filter may actually hold back. However, the fact that the filter may impair the performance depends on the driving behavior of the subject, as can be seen in Figure 11, when we compare day 2 with day 3. Both days show almost the same performance without filter, but the performance *with* filtering is different. The difference may be attributed to a change in driving behavior. In the detail of Figure 12, for instance, we can see that the subject tries to turn 180 degrees in a corridor, behavior which is not deemed likely by the filter (remember that the filter assumes the intention of smooth and efficient *forward* motion). Because it is not deemed likely, many of the subject's steering commands during this manoeuvre are filtered out, which explains the decrease in classifier performance. During day 2 (from which Figure 12 was taken), subject 2 supposedly was still exploring the details of the driving model of the system *with* environmental filter and hence he tried some steering that is incompatible with the filtering assumptions. On day 3, manoeuvres as the one shown in Figure 12 were less in number, supposedly because the mental model that the subject had of the system was more mature by then. All in all, Figure 10 shows that the filter keeps the performance (on the individual command level) more or less constant over all days, roughly between 61% and 69%, in contrast with the more variable decision performance when no filtering is used. Over all sessions and days, the environmental filter improved the individual decision performance with 7.25% for subject 1 and 7.70% for subject 2.

#### 4.3.2. The task level

**Figure 13:**
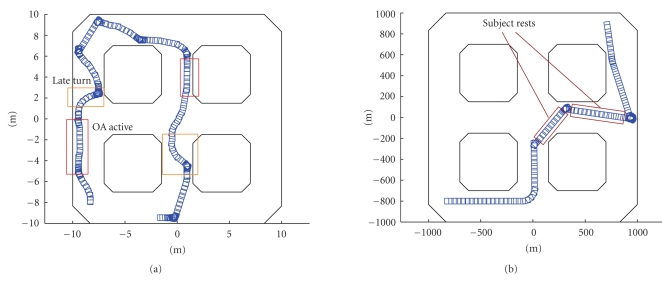
Typical problems that occur while driving. On the left, a path is shown that is driven without the environmental filter. We can see that there are many near collisions (obstacle avoidance gets active), resulting in a rather jagged path. On the right a session with filtering is shown. It is clear that the overall path is more smooth, although near collisions still occur (mainly due to inappropriate resting periods).

Interesting in itself, the results obtained on the individual command level do not reflect the *driving behavior* . Even if the speeds that are sent to the motors are *on average* very much what the subject wants them to be, that does not necessarily result in good driving behavior. Much more is involved when controlling mobile robots such as the wheelchair. For one, *timing* is critical. When the corner arrives, the steering needs to be correct *at that very moment* , not just on average over the whole session. Also, the human needs to have good understanding of the kinematic and dynamical constraints of the robot, to predict its movement and hence correctly time the steering. To get a qualitative feeling of the typical driving problems that may occur, see Figure 13. It is clearly visible at Figure 13(a) that steering commands may arrive rather late, when the opportunity of turning into the corridor has already passed. Two main causes underlie this behavior. On the one hand, the subject's kinematic insight is impaired by the large mental workload that the fine steering requires. Therefore, the commands for turning may be generated too late or too soon. On the other hand, switching directions (i.e., from *Forward* to *Right*) always takes some time, because the user has to shift his/her thoughts to another mental task to generate another steering signal. While this switching is occurring, the wheelchair simply drives on and critical moments are passing by. Figure 16 schematically shows this process. Also visible is that a fair amount of “wall following” is occurring, that is, the subject gets too close to a wall and obstacle avoidance is activated, leading the wheelchair alongside the wall. When the subject does not take action to get away from the wall, a large percentage of the session time may be spent in OA mode. This is undesirable, as it results in a reduction of the average velocity and thus in a degraded overall task performance.

When driving with environmental filtering, the path is typically much smoother (see Figure 13(b)). Problems that may occur are that the subject chooses his/her resting periods at inappropriate moments. When driving the wheelchair, resting periods are most appropriate when driving straight on in a corridor. The robot will stay on course. Whenever a choice in the path (e.g., the possibility to turn left or right) arises, however, the subject needs to take control and convey his/her intention to the system. In other words, resting periods cannot be chosen arbitrarily but must be appropriately timed as well. For instance, as shown in Figure 13, the subject takes two long rests, right at the moment when he/she needs to decide over the general direction to take. This behavior has a negative impact on the smoothness of the path and the resulting average velocity.

**Figure 14:**
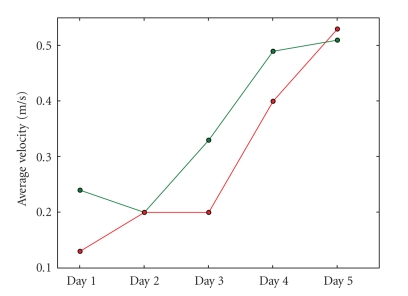
The evolution of the average velocity during sessions over all five days for subject 1. The lower line represents the performance when driving without filter, the upper one the average velocity when the filter is active. It is clear that the overall performance (with and without filter) improves significantly over the course of days.

**Figure 15:**
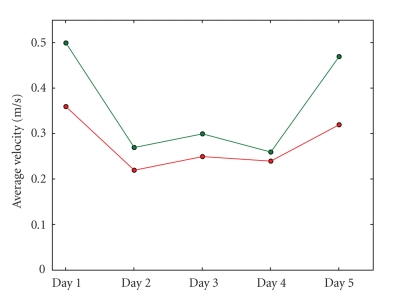
The evolution of the average velocity during sessions over all five days for subject 2. The lower line represents the performance when driving without filter, the upper one the average velocity when the filter is active. We can see that the average velocities are much higher when driving with filtering, especially during the first and last days.

It is also noteworthy to mention that the overall average velocity for subject 1 rises over the days as Figure 14 shows, indicating that the subject's driving skills improve gradually. Subject 2 does not show a similar evolution (see Figure 15), but *in both cases* we can see that the average velocities are much higher when filtering is active. For subject 1, the average improvement the filter offers regarding the average velocity is 17.58%. For subject 2 the gain is even higher: 22.72%.

Another interesting factor is the time the user spent in obstacle avoidance mode, as this reflects an undesirable aspect: namely that the subject is controlling the robot to a lesser extent when in this mode. Furthermore, OA is designed as a safety measure, not to provide continuous navigational assistance. All in all, spending much time in OA does not constitute what we regard as “good driving behavior” and it slows the navigation down significantly. When we compare the average amount of time spent in OA mode when driving without filter to the amount when driving with environmental filtering, we see an overall 13.3% (subject 1) and 17.44% (subject 2) decrease for the latter case. This is reflected in the more efficient (centre of corridor) driving behavior.

Now, if we consider the task that the subjects had to perform (driving to a certain goal pose, if possible via a predetermined path, as shown in Figure 6) we get the results for subject 1 listed in Table 1. This table shows the figures for day 2 through 5 (there was no goal-directed task in day 1). It is clear that for the majority of days, the environmental filter significantly increases the probability of reaching the goal position within 4 minutes. Only during the last day, the subject had better control without the filter and could reach the goal 100% of the time. All sessions together, the filter proves to increase the task performance by about 10%. Considering only days 2 through 4, we see an increase of more than 26%. Subject 2 on the other hand, did not show a large difference in probability of reaching the goal with or without filtering (+7.5% when filtering is active). However, the *time* needed to reach the goal is significantly lower when using the environmental filter, as Table 2 shows. In total, subject 2 reached the goal 19.87% more rapidly when driving with filter compared to driving without.

## 5. DISCUSSION

**Figure 16:**
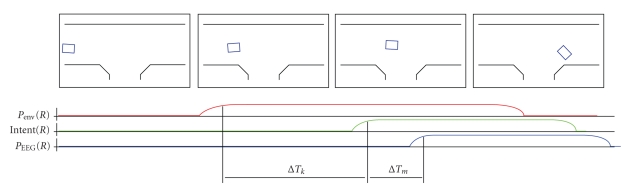
The timing problem. The upper row of figures shows a few frames in the path a subject may follow when the task is to turn south into the *T* -shaped corridor. We can see the evolution of the probabilities for *Right* commands as seen from the environmental filter and the BCI. Also visible is the moment at which the user decides to start turning, shown as *(R)*. The timing issue has 2 components. First, there is a delay δ*T_k_* that denotes the suboptimal kinematic prediction by the subject; the turn should have started earlier for the current speed of the platform. Secondly, a mental task switching delay *T_m_* occurs which increases the total time delay even more. Eventually, the opportunity has passed by and the wheelchair crashes into the wall.

The experiments were conducted with two subjects that had no previous experience in BCI control nor control of differentially driven robots such as the electrical wheelchair. From the data collected over all five days, we can clearly see how the subjects have gradually learned to improve this control. Yet, the problem of predicting the kinematic behavior of the wheelchair accurately remains a mentally demanding task. This may cause erroneous timing when initiating a turn into a corridor, leading to a worse overall driving behavior; refer to Figure 16 for a graphical overview of this problem.

During the course of the first days, when the subjects were still performing rather bad, the filter acts as a correctional tool that rectifies much of the misclassifications coming from the EEG classifier. This is visible in Figures 10, 11, 14, 15 and Table 1. As a result, filtering enables a novel subject to achieve good levels of performance even on the *first* day of usage. It is clear from Figures 10 and 11 that the filter keeps the performance on the level of the individual commands more or less stable over all days. The environmental filter may thus be seen as a *learning tool* that keeps the subjects performance on a workable level even if that subject is just taking the first steps in learning to use BCI control for driving a wheelchair. Later on, when the subject shows an improved control, the filter corrects less, up to the point that the control is so good that the filter actually holds back. It is remarkable that on the first day, when the subjects still were completely new to the task, for some (filtered) sessions very good performance could be noted.

However, the collected data and the performance figures extracted from the experiments are to a large extent dependent on the *driving strategy* the subject employs. As the subjects gradually learned to control the system, different strategies were explored. One example is visible in Figure 13(a), where the subject exploited the behavior provided by the obstacle avoidance algorithm to lead the robot without much control effort alongside a wall. Similarly, the subject occasionally exploited the OA safety behavior to let the robot ride until it approaches a wall. At that point, OA slows the robot down and the subject has more time to choose the direction he/she wants to go into. This is, for instance, visible in Figure 13(b). Now, while exploring alternative strategies, the performance measures naturally change as well.

A further source of “noise” on the collected data is caused by inappropriate usage of the resting possibility, as already discussed before. Figure 13-right shows an example. Of course, this strategy also has a negative influence on the resulting performance.

Furthermore, the filter was regularly switched on and off in between sessions, without the subject's knowledge. Because of the fact that the driving system is different when the filtering is applied, the subject needs to use another mental model (or at least adapt his/her existing one) when the filter is switched on or off. Also, the subjects were not told how the environmental filter internally works, so that they needed to learn an appropriate mental model from scratch while driving. The result is that when the subject's acquired strategies built up using the one driving system (i.e., without filtering) were applied to the other situation, performance was seriously weakened. This effect is sometimes referred to as *mode confusion* [14] and it is a common problem in shared control systems. An illustrative example is that when driving without filtering, the subjects learned at a certain moment to turn 180 degrees in a corridor, whenever they got orientated in the wrong direction (see Figure 12). When the filter was switched on, he/she tried to use that same strategy. Because the filter assumes smooth and efficient forward motion, such behavior was deemed unlikely and the filter made it a difficult manoeuvre. This leads to a situation in which the environmental filter is actually working *against* the user's intention.

## 6. CONCLUSIONS AND FURTHER WORK

We have shown that the usage of an environmental filtering technique, which uses knowledge about the current context to filter out erroneous steering commands, can improve the overall driving behavior. Especially when the subject is not already trained for the task, the filter provides significant benefits. However, when the subject is performing really well and employs driving behavior that is not compatible with the logic of the filter, performance may be weakened. All in all, the subjects declared that driving with filtering was *more easy* to do, especially during the first days. As such, the system proves most useful as a learning tool, when the subject is in the learning phase of BCI control.

Probably the most notable weakness of the filter in its current form is the fixed user model. The system assumes a certain driving behavior that would lead to smooth and efficient forward motion. Whenever strategies are employed that contradict with this assumption, the performance gets worse (i.e., 180-degree turning in a corridor). Therefore, we need an *adaptive* model that constantly adapts to whatever strategies the user might employ. Besides that, we could also benefit from a detection mechanism that simply switches off the filter if user performance gets high, or more generally some mechanism to regulate the amount of influence the filter has. Also, a user model incorporating the specific BCI profile the particular subject has (how likely it is that he/she generates the correct steering commands) might lead to a better filtering of the individual commands.

## Figures and Tables

**Table 1:** The percentage of sessions in which subject 1 reached the goal position within 4 minutes.

Day	Overall (all sessions)	Sessions without filtering	Sessions with filtering
Day 2	60%	40%0	80%
Day 3	80%	66.67%	85.71%
Day 4	70%	60%	80%
Day 5	80%	100%	60%

**Table 2:** The time subject 2 needed to reach the goal position (in s).

Day	Overall (all sessions)	Sessions without filtering	Sessions with filtering
Day 2	151.32	164.25	138.4
Day 3	120.37	144.6	115.52
Day 4	138.7	145.6	127.2
Day 5	110.26	126.01	84.01

## Footnotes

http://www.mech.kuleuven.be/mlr.
